# Supplementary material for: A Critical View of the Application of the APEX Software (Aqueous Photochemistry of Environmentally-Occurring Xenobiotics) to Predict Photoreaction Kinetics in Surface Freshwaters
Source: Molecules. 2019 Dec 18;25(1):9. doi: 10.3390/molecules25010009 (PMC7017383; doi:10.3390/molecules25010009)
Supplement: Supplementary file 1 [file molecules-25-00009-s001.zip › 3.2.4_gcc-4.4.0.zip!/3.2.4_gcc-4.4.0/3.2.4_gcc-4.4.0/doc/octave/html/interpreter/Structures-in-Oct_002dFiles.html]

Structures in Oct-Files - Untitled


Next: Sparse Matrices in Oct-Files,
Previous: Cell Arrays in Oct-Files,
Up: Oct-Files


---

#### A.1.5 Structures in Oct-Files

A structure in Octave is map between a number of fields represented and
their values. The Standard Template Library `map` class is used,
with the pair consisting of a `std::string` and an octave
`Cell` variable.

A simple example demonstrating the use of structures within oct-files is

```
```
     #include <octave/oct.h>
     #include <octave/ov-struct.h>
     
     DEFUN_DLD (structdemo, args, , "Struct demo.")
     {
       int nargin = args.length ();
       octave_value retval;
     
       if (nargin != 2)
         print_usage ();
       else
         {
           Octave_map arg0 = args(0).map_value ();
           std::string arg1 = args(1).string_value ();
     
           if (! error_state && arg0.contains (arg1))
             {
               // The following two lines might be written as
               //    octave_value tmp;
               //    for (Octave_map::iterator p0 = 
     	  //        arg0.begin(); 
               //        p0 != arg0.end(); p0++ )
               //      if (arg0.key (p0) == arg1)
               //        {
               //          tmp = arg0.contents (p0) (0);
               //          break;
               //        }
               // though using seek is more concise.
               Octave_map::const_iterator p1 = arg0.seek (arg1);
               octave_value tmp =  arg0.contents(p1)(0);
               Octave_map st;
               st.assign ("selected", tmp);
               retval = octave_value (st);
             }
         }
       return retval; 
     }
```
```

An example of its use is

```
     x.a = 1; x.b = "test"; x.c = [1, 2];
     structdemo (x, "b")
     => selected = test
```

The commented code above demonstrates how to iterate over all of the
fields of the structure, where as the following code demonstrates finding
a particular field in a more concise manner.

As can be seen the `contents` method of the `Octave_map` class
returns a `Cell` which allows structure arrays to be represented.
Therefore, to obtain the underlying `octave_value` we write

```
     octave_value tmp = arg0.contents (p1) (0);
```

where the trailing (0) is the () operator on the `Cell` object. We
can equally iterate of the elements of the Cell array to address the
elements of the structure array.
